# Investigating MicroRNA and transcription factor co-regulatory networks in colorectal cancer

**DOI:** 10.1186/s12859-017-1796-4

**Published:** 2017-09-02

**Authors:** Hao Wang, Jiamao Luo, Chun Liu, Huilin Niu, Jing Wang, Qi Liu, Zhongming Zhao, Hua Xu, Yanqing Ding, Jingchun Sun, Qingling Zhang

**Affiliations:** 1 Department of Pathology, Nanfang Hospital, Southern Medical University, Guangzhou, 510515 China; 2 Department of Pathology, College of Basic Medicine, Southern Medical University, Guangzhou, 510515 China; 3 Center for Quantitative Sciences, Vanderbilt University School of Medicine, Nashville, TN 37232 USA; 4 School of Biomedical Informatics, The University of Texas Health Science Center at Houston, Houston, TX 77030 USA; 5 Center for Precision Health, School of Biomedical Informatics, The University of Texas Health Science Center at Houston, Houston, TX 77030 USA

**Keywords:** Colorectal cancer (CRC), microRNA, Transcription factor, Feed-forward loops (FFLs), Regulatory network

## Abstract

**Background:**

Colorectal cancer (CRC) is one of the most common malignancies worldwide with poor prognosis. Studies have showed that abnormal microRNA (miRNA) expression can affect CRC pathogenesis and development through targeting critical genes in cellular system. However, it is unclear about which miRNAs play central roles in CRC’s pathogenesis and how they interact with transcription factors (TFs) to regulate the cancer-related genes.

**Results:**

To address this issue, we systematically explored the major regulation motifs, namely feed-forward loops (FFLs), that consist of miRNAs, TFs and CRC-related genes through the construction of a miRNA-TF regulatory network in CRC. First, we compiled CRC-related miRNAs, CRC-related genes, and human TFs from multiple data sources. Second, we identified 13,123 3-node FFLs including 25 miRNA-FFLs, 13,005 TF-FFLs and 93 composite-FFLs, and merged the 3-node FFLs to construct a CRC-related regulatory network. The network consists of three types of regulatory subnetworks (SNWs): miRNA-SNW, TF-SNW, and composite-SNW. To enhance the accuracy of the network, the results were filtered by using The Cancer Genome Atlas (TCGA) expression data in CRC, whereby we generated a core regulatory network consisting of 58 significant FFLs. We then applied a hub identification strategy to the significant FFLs and found 5 significant components, including two miRNAs (hsa-miR-25 and hsa-miR-31), two genes (*ADAMTSL3* and *AXIN1*) and one TF (BRCA1). The follow up prognosis analysis indicated all of the 5 significant components having good prediction of overall survival of CRC patients.

**Conclusions:**

In summary, we generated a CRC-specific miRNA-TF regulatory network, which is helpful to understand the complex CRC regulatory mechanisms and guide clinical treatment. The discovered 5 regulators might have critical roles in CRC pathogenesis and warrant future investigation.

**Electronic supplementary material:**

The online version of this article (10.1186/s12859-017-1796-4) contains supplementary material, which is available to authorized users.

## Background

Colorectal cancer (CRC) is one of the most common malignant tumors in the human digestive system and has the third highest incidence and mortality of all malignancies [1–3]. Uncovering the regulation and progression mechanisms of CRC is important for developing effective molecular therapeutic strategies. In the last decades, substantial efforts have been made to collect samples and generate the data, from which the findings have greatly improved our understanding of the molecular basis of cancers; these efforts include genomic profiling analysis of cancer such as large-scale genome sequencing projects [4–6]. The Cancer Genome Atlas (TCGA), one of the largest cancer-related genome analysis projects, contributed many impellent effects to the understanding of the underlying genetics of CRC, such as mutation characteristics and copy number alterations [7–9]. Moreover, there were several genome-wide analyses which greatly contributed to the comprehensive profiling of CRC whose results provided significant evidence for the association between loci or genes and CRC. These included single nucleotide polymorphisms (SNPs) in genes encoding SMAD7, laminin gamma 1, T-box 3, cyclin D2, etc. [10–13]. These studies have demonstrated that there are many genetic and epigenetic alterations in one or several processes simultaneously. Although these findings seemed not so systematical to reveal an intuitive concept for the biological process of CRC, it provided a hint that a comprehensive method should be used to uncover the underlying regulation mechanism of these bio-molecules.

Network analysis, such as feedback loop (FBL) and feed-forward loop (FFL), is a powerful way to investigate the underlying global topological structures of molecular networks [14–17]. miRNA-transcription factor (TF) co-regulation is one of the important FFL type. Building and mining miRNA-TF co-regulation networks served as a valuable approach to investigate the cell regulation in many systems and cell types, including various kinds of cancers [17–19]. miRNAs are evolutionarily conserved, endogenous, small, and noncoding RNAs molecules of about 22 nucleotides in length. miRNAs play important roles in post-transcriptional gene regulation during the initiation and progression of human cancers [20–23]. A spectrum of dysregulated miRNAs were also identified between CRC and normal colorectal tissues [24]. For example, over expression of miR-20a and weak expression of miR-133b have been consistently reported in CRC versus normal tissues, and play crucial roles in both metastasis and survival [25–28]. TFs regulate gene expression through translating cis-regulatory codes into specific gene-regulatory events. Accompanied with miRNAs, TFs participate in the regulatory network that controls thousands of mammalian genes [14]. Through the co-regulation model, miRNA and TF regulate their mutual target genes: miRNAs regulate gene’s post-transcription through binding the 3′ untranslated region (UTR) while TFs regulate gene’s transcriptions through binding to the gene’s promoter region [29]. Additionally, TF can regulate miRNA, or to be regulated by miRNA, so that the relationships among miRNAs and TFs and their shared targets form a diversity of feed-forward loops (FFL) [14]. The typical mixed FFL motif defined as a 3-node FFL consists of three components: TF, miRNA and their mutual regulated gene. Recently, FFL-based combinatorial regulatory network approach has emerged as a promising tool to elucidate complex diseases, such as schizophrenia [30], glioblastoma multiforme [31, 32], ovarian cancer [33], lung cancer [34], and osteosarcoma [35]. However, network based on 3-node FFLs has not been established in CRC, one of the common cancers.

In this study, we investigated the comprehensive miRNA-TF co-regulatory network in CRC through modifying the well-developed framework in our previous studies [32, 33]. Among the candidate genes, we identified the potential targets of CRC-related TFs and miRNAs, then built a comprehensive CRC-specific miRNA-TF mediated regulatory network. Finally, we divided this massive network into three subnetworks on the basis of their inside regulatory relationships, followed by a topology analysis. However, such regulations might include some false positives due to the limitation to recent regulatory prediction databases.

The TCGA studies generated vast quantities of gene expression profiling and other molecular profiling from hundreds of CRC samples, which provide the promising opportunity to uncover the basic building blocks of regulatory networks in CRC [9]. Thus, compared to our previous methods [32, 33], we took the advantage of the gene and miRNA expression data in CRC patients from TCGA project to improve the accuracy of the results [7, 9]. This integration with experimental data from patients is a complement to the FFL studies which mostly relied on the predicted regulation information by reducing false positives. After these systematic analysis, we identified six hub components. To verify the implication of these components, we further explored the associations between the expression level of identified components and CRC survival. This study established a valuable CRC progress regulation network, which can provide information about further experimental exploration and help to reveal the complicated regulatory mechanisms and find out new markers or targets for the diagnoses and treatments for CRC.

## Methods

### CRC-related genes and miRNAs

**Fig. 1:**
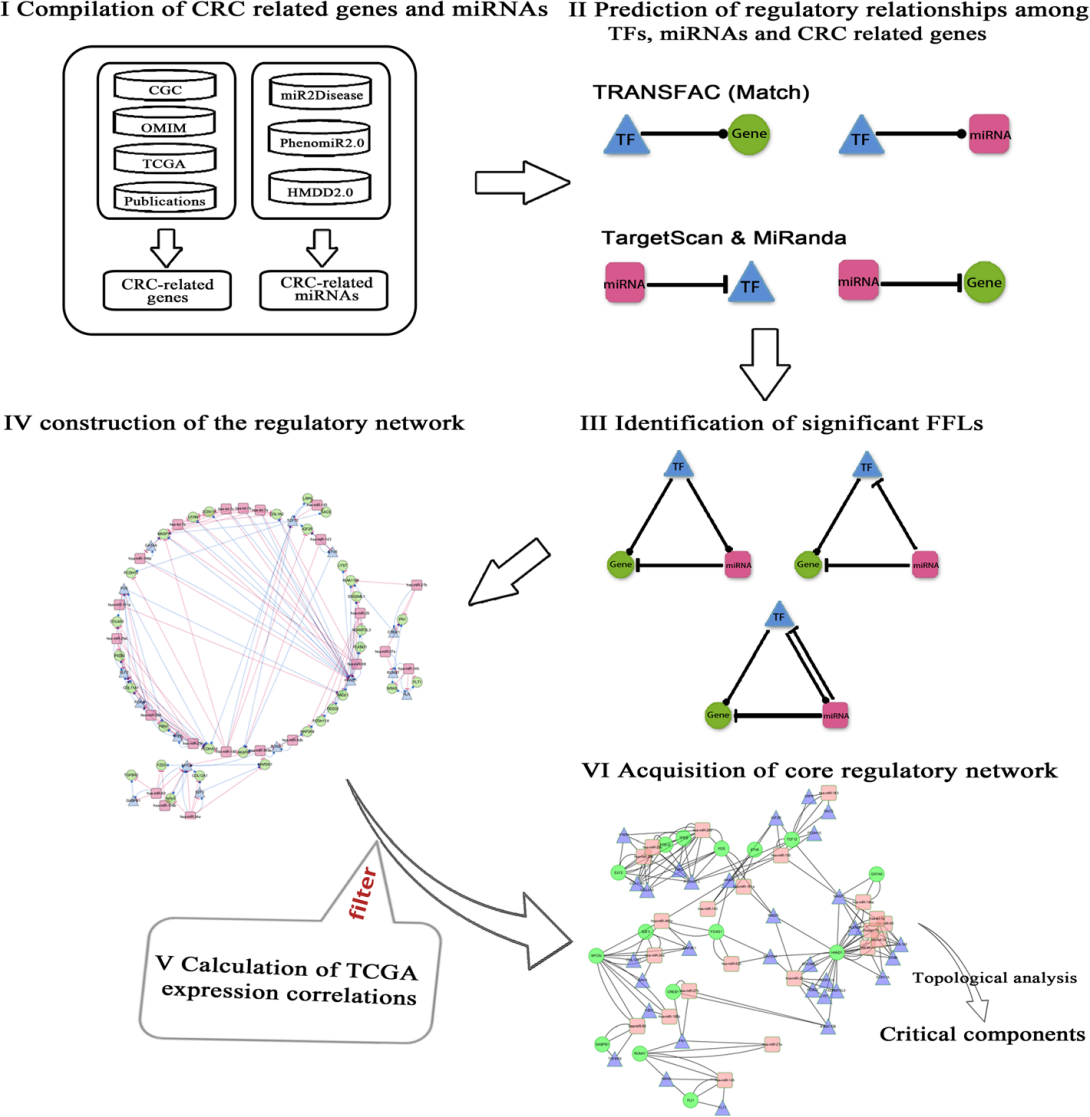
Process of miRNA-TF regulatory network construction and significant FFLs identification in colorectal cancer (CRC). This process contains six steps. 1) Data compilation. We extracted CRC-related genes, CRC-related microRNAs (miRNAs), and human transcription factors (TFs) from multiple databases. 2) Prediction of the regulatory relationships. The four regulatory relationships include TF-gene, TF-miRNA, miRNA-gene, miRNA-TF. 3) Feed-forward loop identification. Based on the regulatory relationships above, the significant 3-node feed-forward loops were identified. 4) CRC-specific miRNA-TF regulatory network construction and further analysis by merging the FFLs identified in step three. 5) TCGA expression correlation calculation. We calculated the expression correlations of each pair in the network, and removed the false positive pairs. 6) Acquisition of significant FFLs. We extracted the core subnetwork based on the significant pairs identified in step five. Furthermore, identification of critical miRNA and gene components were performed

We collected CRC-related genes from five sources (Fig. 1). These sources included the Cancer Gene Census (CGC, available at [36]), the Online Mendelian Inheritance in Man (OMIM, available at [37]), The Cancer Genome Atlas (TCGA) publication [9] and its mutation data (available at [38]), and a mutation landscape research [39]. Finally 464 unique genes were obtained (Additional file 2: Table S1 and Additional file 3: Text S1).

To obtain the dysregulated miRNAs in CRC, we searched the miR2Disease (available at [40]), PhenomiR2.0 (available at [41]), and HMDD2.0 (available at [42]) by using the keywords “colorectal cancer” or “colorectal neoplasms or colonic neoplasms”. The expressions of miRNAs obtained from miR2Disease and PhenomiR2.0 have already been recorded. For HMDD2.0, we downloaded the full papers through the related PubMed ID and read those texts to identify the expression comparison between CRC and normal controls. Finally, 257 unique miRNAs were retrieved as CRC-related miRNAs (Additional file 2: Table S2 and Additional file 3: Text S2).

### Prediction of the regulatory relationships

We applied the TargetScan and the miRanda to obtain the regulatory relationship between miRNAs and CRC-related genes or human TFs. We downloaded the TargetScan database (Release 6.2, available at [43]) and extracted the miRNA-gene pairs. These pairs are evolutionarily conserved in the four species (include human, mouse, rat and dog) and have a total context score higher than −0.30. For miRanda (available at [44]), we extracted the target pairs conserved in human, mouse and rat with the condition of S > 90 and ΔG < −17. Then we merged the two sets of miRNA-gene pairs together. To obtain the regulation of miRNA to TF, we retrieved 1201 TFs from the TRANSFAC Professional Database (release 2011.4) [45]. We extracted the TFs based on its CRC-related target promoter region sequences (−1500/+500 around TSS). Then we performed a binding sites search of TFs to the defined promoter region of the CRC-related targets. Then we used pre-calculated cut-offs to minimize false positive (minFP) matches and created a high-quality matrix. To restrict the search, we required a core score of 1.00, a matrix score of 0.95, and TF that only belong to the human genome. To further reduce false positive prediction, we required the predicted pairs to be conserved among humans, mice and rats. For the regulation of TF to genes/miRNAs, we followed the procedure we utilized in our previous work [32].

### Selection of significant regulations based on TCGA expression data

The Cancer Genome Atlas (TCGA) project provides a large data to the cancer research. We first downloaded the CRC-related expression data from the TCGA Data Portal (available at [38]), and calculated the correlation among the gene and miRNA nodes of the regulatory networks. Significant pairs were selected on the basis of the expression Pearson correlation coefficient (R). For TF-gene pairs, we required *R* ≥ 0.14 or *R* ≤ −0.14 (adjusted *P*-value <0.01, adjusted by FDR, one-tailed probability, sample size = 264). For miRNA-gene pairs, we required *R* ≤ −0.15 (adjusted *P*-value <0.01, adjusted by FDR, one-tailed probability, sample size = 243). For TF-miRNA pairs, we required *R* ≥ 0.15 or *R* ≤ −0.15 (adjusted *P*-value <0.01, adjusted by FDR, one-tailed probability, sample size = 243). For miRNA-TF pairs, we required *R* ≤ −0.15 (adjusted *P*-value <0.01, adjusted by FDR, one-tailed probability, sample size = 243).

### Significant component expression and survival correlation analysis

Expression and survival data was obtained from the OncoLnc database, available at [46]. The optimum cutoff level of expression of each component was selected on the basis of the association with the patients’ survival by using a tool X-tile (version 3.6.1). A log-rank test was used to compare survival curves.

### Network visualization and data analysis

We visualized the network by using Cytoscape (version 3.2.0) [47]. All statistical analyses were performed with R software and appropriate packages, available at [48].

## Results

### Regulatory relationships among miRNAs, TFs, and genes

To build miRNA-TF co-regulatory networks in CRC, we modified the computational framework developed in our previous studies (Fig. 1). In the process, the 464 CRC-related genes with mutation evidence from five data sources (Additional file 2: Table S1 and Additional file 3: Text S1), the 257 miRNAs that reported to be dysregulated in the CRC (Additional file 2: Table S2 and Additional file 3: Text S2), and the 1201 TFs from TRANSFAC Professional (release 2011.4) [49] were collected. 1201 TFs were not preselected based on other evidences related to CRC, but filtered out by strict requirements when identified regulatory (see Methods). Four types of regulatory relationships among genes, miRNAs and TFs were predicted by using the methods described in our previous study [32]. Prediction results of the regulatory relationships were summarized in Table 1. These predicted relationships were named as prediction data.

**Table 1:** Regulatory relationships among CRC-related genes, CRC-related miRNAs and TFs

Relationship	Number of pairs	Number of miRNAs^a^	Number of genes	Number of TFs^b^	Method
miRNA-gene^c^	201	60	91	-	TargetScan and miRanda
miRNA-TF^d^	106	58	-	43	Match™
TF-gene^e^	42,023	-	401	189	Match™
TF-miRNA^f^	25,109	234	-	189	Match™
Total	67,439	235	410	189	-

^a^miRNA: microRNA

^b^TF: transcription factor

^c^miRNA-gene: miRNA repression of gene expression

^d^miRNA-TF: miRNA repression of gene expression

^e^TF-gene: TF regulation of gene expression

^f^TF-gene: TF regulation of miRNA expression

### CRC-specific regulatory networks generated from prediction data

By merging the regulatory relationships predicted above, 3-node FFLs were formed (Table 2). The 3-node FFL, as one of the most common types of motifs in transcriptional network, can be classified into three categories: miRNA-FFL, TF-FFL and composite FFL, which are based on their inside regulations and have been described in our previous study [32]. In general, in miRNA-FFL, the miRNA represses both TF and gene expression while the TF regulates target gene expression. In TF-FFL, the TF regulates the miRNA and the gene while the miRNA represses the target gene. In composite-FFL, the TF regulates the miRNA and target gene while the miRNA represses the TF and the gene. The three types of FFLs are exclusive to each other.

**Table 2:** Summary of 3-node feed-forward loops based on CRC-related prediction data

Number of nodes^a^					Number of links				
3-node motif	Number of merged FFLs^b^	Genes	miRNAs	TFs	Total	TF-gene	miRNA-gene	miRNA-TF	TF-miRNA
TF-FFL	13,005	82	59	170	12,680	7001	174	0	5505
miRNA-FFL	25	20	13	12	61	23	23	15	0
Composite-FFL	93	42	30	24	225	64	77	42	42
Total	13,123	82	59	171	12,821	7043	174	57	5547

^a^Definition of the nodes and links is the same as in Table 1

^b^FFL: feed-forward loop

**Fig. 2:**
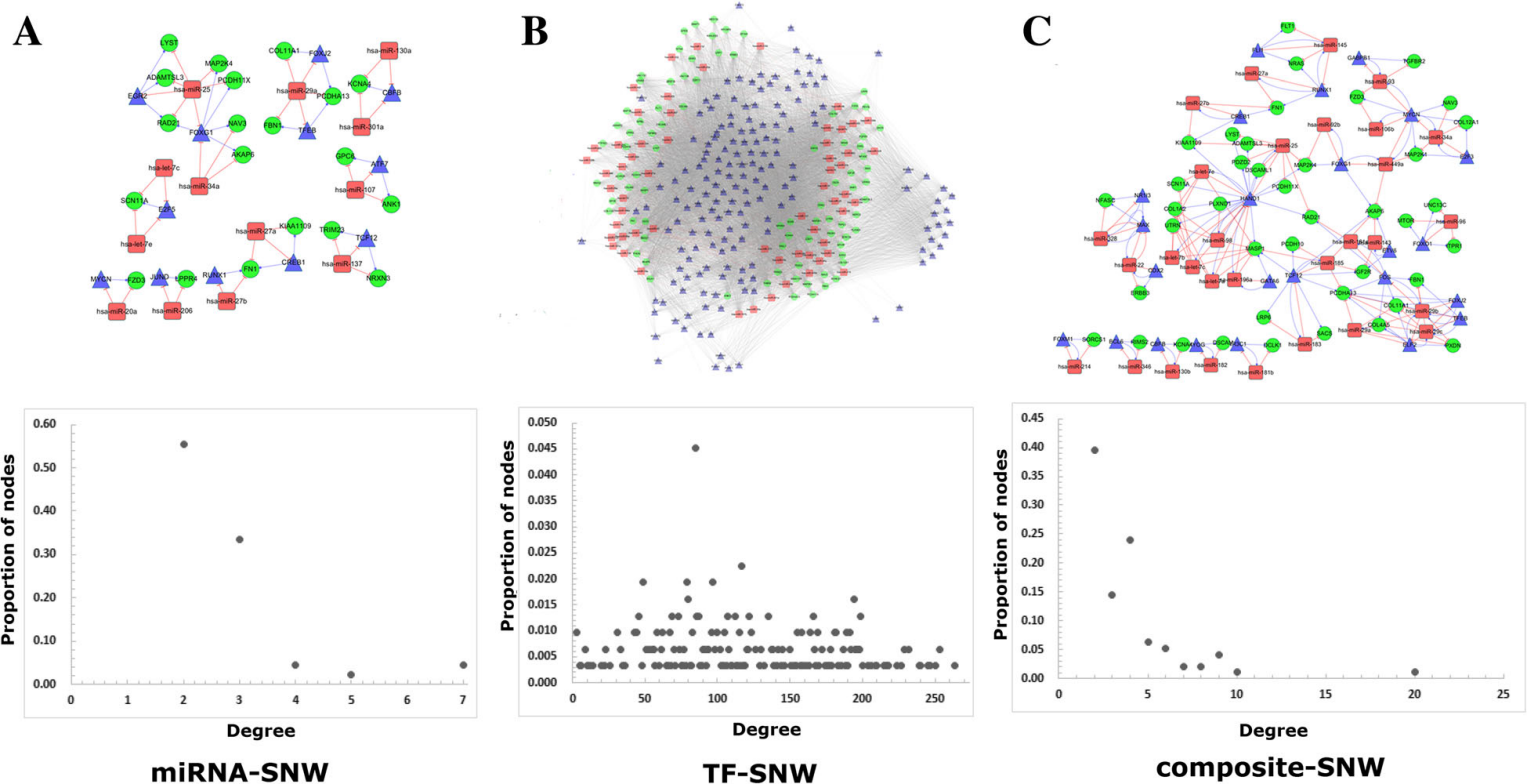
Graphical representations of three types of CRC-specific regulatory subnetworks. **a**) miRNA-SNW. This subnetwork was constructed by miRNA-FFLs, including three types of regulatory relationships: miRNA-TF, miRNA-Gene, TF-Gene. **b**) TF-SNW. The subnetwork was constructed by TF-FFLs, including three types of regulatory relationships: TF-miRNA, TF-Gene, miRNA-Gene. **c**) composite-SNW. This subnetwork was constructed by composite-FFLs, including four types of regulatory relationships: TF-miRNA, miRNA-TF, TF-Gene, miRNA-Gene. In three subnetworks, the node colors represent different molecules: red for CRC-related miRNAs, blue for transcription factors, and green for CRC-related genes. Edges in red correspond to the repression of miRNAs to genes or TFs, and edges in blue correspond to the regulation of TFs to genes or miRNAs. Scatter plots below the networks show the degree distributions of all nodes in 3 kinds of CRC-specific regulatory networks

A miRNA-TF mediated network was constructed for CRC based on 3-node FFLs obtained above. The network contained 12,821 edges and 312 unique nodes of the 13,123 FFLs (Additional file 2: Table S3). Among the 12,821 edges, 174 were miRNA-gene pairs, 57 were miRNA-TF pairs, 7043 were TF-gene pairs, and 5547 were TF-miRNA pairs. Among the 312 nodes, 82 were CRC-related genes, 59 were CRC-related miRNAs, and 171 were human TFs. Considering that these FFLs could be categorized into miRNA-FFLs, TF-FFLs, and composite-FFLs, three subnetworks consisted of corresponding type of FFL were generated accordingly. We named them miRNA-SNW, TF-SNW, and composite-SNW, respectively (Fig. 2). To provide a general view of them, we calculated the degrees and their distributions in all the three subnetworks [50].

The miRNA-SNW composed of 25 (25 out of 13,123, 0.19%) miRNA-FFLs containing 61 edges and 45 individual nodes (Fig. 2a and Additional file 2: Table S4). Among the 61 edges, 23 were miRNA-gene pairs, 15 were miRNA-TF pairs, and 23 were TF-gene pairs. Among the 45 nodes, 20 (20 out of 82, 24.39%) were CRC-related genes, 13 (13 out of 59, 22.03%) were CRC-related miRNAs, and 12 (12 out of 171, 7.02%) were human TFs. The degree values for genes, miRNAs, TFs in this network were in the range of 2–4, 2–7, and 2–7, respectively. Especially, the degree distribution for miRNAs was strongest right-skewed. The distribution pointed out that most of the nodes had low degrees (less than or equal to 3), while only a small portion of them had high degrees. There was only one miRNA hsa-miR-25 had a high degree value (the degree value was 7) (Fig. 2 and Additional file 2: Table S5). This distribution analysis uncovered that hsa-miR-25 regulated more targets than any other regulators.

The TF-SNW was consisted of 12,680 edges and 311 unique nodes from 13,005 (13,005 out of 13,123, 99.10%) TF-FFLs (Fig. 2b and Additional file 2: Table S4). Among the 12,680 edges, 174 were miRNA-gene pairs, 7001 were TF-gene pairs, and 5505 were TF-miRNA pairs. Among the 311 nodes, 82 (82 out of 82, 100%) were CRC-related genes, 59 (59 out of 59, 100%) were CRC-related miRNAs, and 170 (170 out of 171, 99.42%) were human TFs. The degree values of genes, miRNAs, TFs ranged from 44 to 191, 97 to 264, and 3 to 200, respectively. However, their degrees followed a normal distribution. This means that there were few extreme values and was not as helpful as the other two subnetworks for finding biologically critical nodes (Fig. 2 and Additional file 2: Table S5).

In the composite-SNW, there were 93 (93 out of 13,123, 0.71%) composite-FFLs, 96 unique nodes, and 225 edges (Fig. 2c and Additional file 2: Table S4). Among the 225 edges, 77 were miRNA-gene pairs, 42 were miRNA-TF pairs, 64 were TF-gene pairs, and 42 were TF-miRNA pairs. Among the 225 nodes, 30 (30 out of 59, 50.85%) were CRC-related miRNAs, 42 (42 out of 82, 51.22%) were CRC-related genes, and 24 (24 out of 171, 14.04%) were human TFs. The result showed that the composite-FFLs occupied pretty low proportion of all the FFLs, while recruited more than half of CRC-related genes and miRNAs. This indicated that the composite-FFLs might play more important roles than the other two kinds of FFLs. In this subnetwork, degree values of genes, miRNAs and TFs ranged from 2 to 10, 2 to 9, and 2 to 20, respectively. The gene that had the largest degree was *MASP1*; and the miRNA and TF having the largest degrees were hsa-miR-25, hsa-miR-29b and HAND1 respectively (Fig. 2 and Additional file 2: Table S5).

Among above three subnetworks, 15 genes (*FZD3*, *KCNA4*, *RAD21*, *KIAA1109*, *LYST*, *SCN11A*, *AKAP6*, *PCDHA13*, *ADAMTSL3*, *PCDH11X*, *MAP2K4*, *COL11A1*, *FBN1*, *NAV3* and *FN1*), 7 miRNAs (hsa-miR-25, hsa-miR-29a, hsa-miR-34a, hsa-let-7c, hsa-let-7e, hsa-miR-27b, hsa-miR-27a) and 8 TFs (FOXG1, TCF12, FOXJ2, MYCN, TFEB, CREB1, RUNX1, CBFB) participated in all subnetworks simultaneously, which suggested that they might act extensively in the CRC regulation. Interestingly, we noticed that hsa-miR-25 had the highest degree value in both of the composite-SNW and miRNA-SNW, suggesting that hsa-miR-25 might be a critical molecule in the regulatory process of CRC.

### CRC-specific significant regulatory network generated by integrating TCGA expression data

The network generated above was systematical and comprehensive, but it was too complicated to explore the specific regulation mechanisms in CRC. To obtain the regulatory relationship with higher accuracy, we took the advantage of the gene and miRNA expression data in CRC patients from TCGA. Firstly, the correlation coefficients among genes, TFs, and miRNAs were calculated, and then stringent constraint conditions (see Methods) were required to define a co-expression. Subsequently, four types of links (miRNA-gene, miRNA-TF, TF-gene, and TF-miRNA) were obtained (Table 3). We named the dataset *Experiment_data* that included all these pairs based on TCGA experimental data.

**Table 3:** Summary of co-expression relationships among CRC-related genes, CRC-related miRNAs, and TFs from TCGA

Co-expression relationship	Number of pairs	Number of miRNAs^a^	Number of genes	Number of TFs^b^
miRNA-gene^c^	550,079	567	19,570	-
miRNA-TF^d^	28,563	553	-	1141
TF-gene^e^	1,126,334	-	19,701	1158
TF-miRNA^f^	60,794	570	-	1201

^a^miRNA: microRNA

^b^TF: transcription factor

^c^miRNA-gene: anti-correlation between miRNA and gene expression

^d^miRNA-TF: anti-correlation between miRNA and TF expression

^e^TF-gene: correlation between TF and gene expression

^f^TF-miRNA: correlation between TF and miRNA expression

**Fig. 3:**
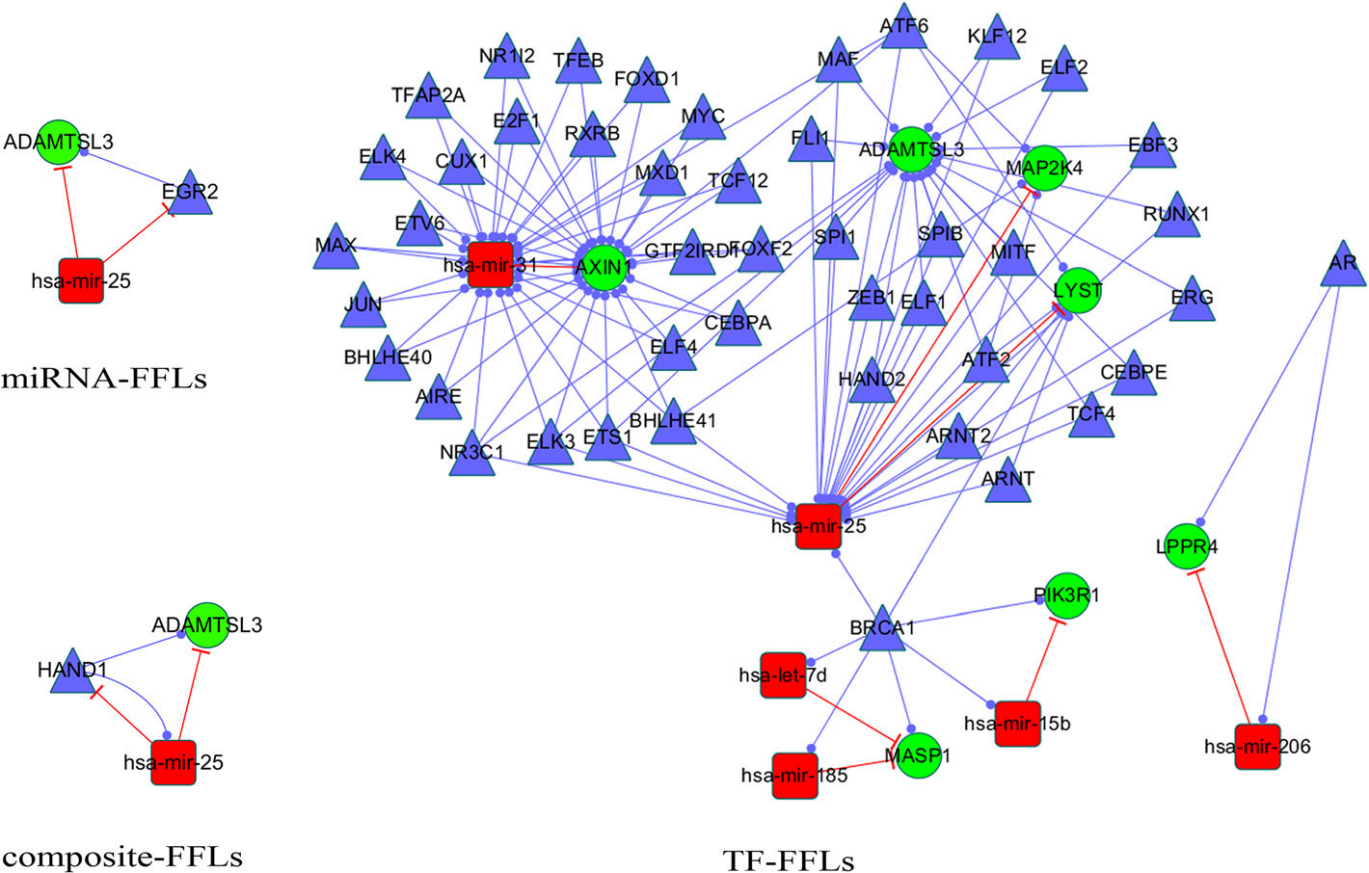
Graphical representation of the significant FFLs. The regulatory network was generated from 3-node FFL motifs common to the prediction data and *Experiment_data*. Shapes and colors definitions for nodes and edges are the same as in the Fig. 2

To reduce the false positives, pairs (regulatory relationships) were required to be conserved in both the prediction data and *Experiment_data*. Finally, one composite-FFL (hsa-miR-25, HAND1, *ADAMTSL3*), one miRNA-FFL (hsa-miR-25, EGR2, *ADAMTSL3*) and 56 TF-FFLs were identified. The regulation details are presented in Fig. 3 ﻿and Additional file 1: Figure S1. The number of TF-FFL was significant more than the other two. In these TF-FFLs, there were 115 edges (55 TF-gene pairs, 53 TF-miRNA pairs, and 7 miRNA-gene pairs) and 58 unique nodes (45 human TFs, 7 CRC-related genes, and 6 CRC-related miRNAs) Additional file 2: Table S6). There are a few nodes exhibited a high degree, which acted as the hubs that might play more important roles in the regulatory networks [51, 52]. Using the hub definition method proposed by Yu et al. [53], we determined the degree cutoff value of 22, 26, and 7 for gene, miRNA and TF hubs respectively (Additional file 2: Table S7). Accordingly, two hub miRNAs (hsa-miR-25 and hsa-miR-31), two hub genes (*ADAMTSL3* and *AXIN1*) and one hub TF (BRCA1) were identified.

### Significant components

As analyzed above, through our consecutive network framework, 5 components were identified, including two hub miRNAs (hsa-miR-25 and hsa-miR-31), two hub genes (*ADAMTSL3* and *AXIN1*) and one hub TF (BRCA1). Such hub identification was mainly based on their degrees in the network. Are these connective characteristics specific to CRC, or just their innate property of the complex regulatory mechanism in our body? We found hsa-miR-25 had more targets (top 5.0%, Additional file 2: Table S8) than most of others miRNAs collected in TargetScan but less targets in miRanda (top 60.0%, Additional file 2: Table S9), and hsa-miR-31 had a moderate number of target in both databases (top 36.8% and 32.8%, respectively). However, some miRNAs, such as hsa-miR-7b and hsa-miR-497, had a high number of targets both in TargetScan (top 4.0% and 0.8%, respectively) and miRanda (top 6.0% and 11.2%, respectively), which were also included in our analysis, but they were not identified as hub nodes after our consecutive analysis. These suggested that the significant miRNA identification was mainly contributed to the regulatory pattern after our regulatory network construction, despite of the relationship distribution and bias in databases might make an impact on the topology of final network.

**Fig. 4:**
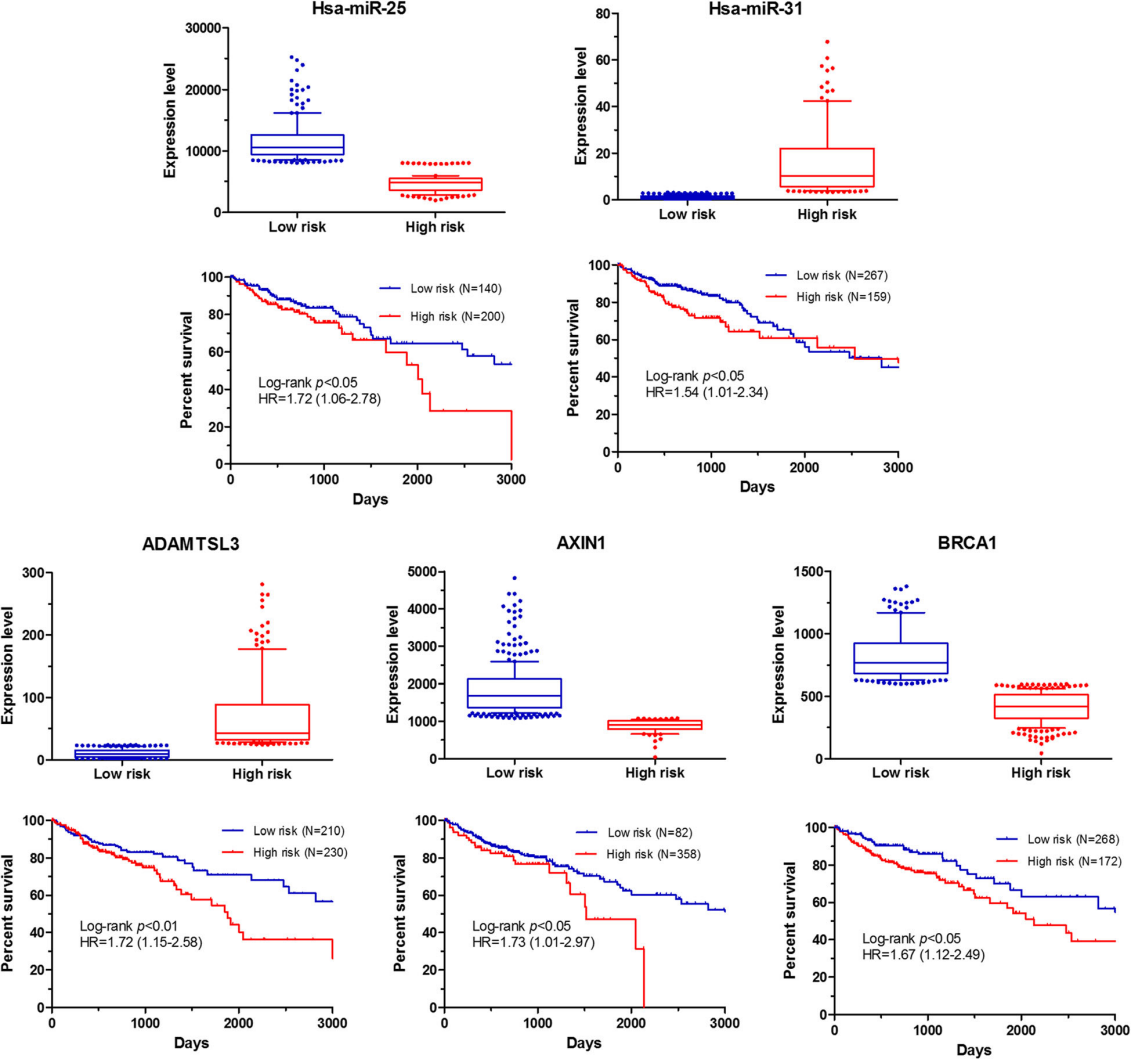
Expression level of significant components and association with overall survival. The expression and survival data for CRC patients was obtained from OncoLnc database. Optimum cut-off level of expression was determined on the basis of their associations with survivals by using X-tile software

To further investigate the implication of the hub miRNAs, TFs and genes for CRC development, we analyzed the correlation between their expression levels and survivals of patients with CRC by using data from OncoLnc database [46]. Figure 4 shows the expression of the significant components in CRC patients with low or high risk to all-caused dead and the survival curves in the low and high risk groups which were identified by the optimal cut-off value of corresponding component expression level. All of the significant components showed a well prediction value for the prognosis of CRC patients. Among 5 significant components, hsa-miR-25, *AXIN1*, ATF6 and BRCA1 exhibited a negative correlation between their expression levels and patients’ survival, while higher expression of *ADAMTSL3* was observed in patients with a better survival. Patients was subdivided well into two groups (namely, low risk and high risk groups) by using these components independently, with significantly different survival curves.

## Discussion

In this study, a co-regulatory network mediated by miRNAs and TFs was first time explored in CRC, one major cancer type. Our results provides some insightful information and a few miRNA and TF candidates, as well as their regulation for further experimental validation in CRC. In this study, our previous computational framework was modified by integrating gene and miRNA expression data from TCGA to improve the result accuracy. We extracted significant components from the whole complex network based on prediction *data* by using the data of *Experiment_data*. Then survival information was used to determine the significant components implication for CRC prognosis.

This unique computational framework has been described in our previous studies [32, 33] and illustrated that it is indeed possible to use a large panel of methods to process multiple types of data (e.g., mutation data, gene expression data, and knowledgebase) to identify potential disease-associated components in complex diseases. To increase the confidence and accuracy in predicting biologically relevant regulations, one strategy is to identify regulatory relationships that are consistent or reproducible in multiple independent studies [54, 55]. In this study, as the major improvement for our previous computational framework, we specifically integrate the prediction data and experiment data in our regulatory network analyses. The experiment data was used to improve the accuracy of results in the prediction data, whereby the significant components were extracted from the whole huge and complex network. So far, such a strategy has not been applied to miRNA-TF co-regulatory network analyses in CRC. Furthermore, with the rapid growth in high-throughput expression profiling studies, this strategy might become not only feasible, but also necessary to identify complex gene regulation in cellular systems and provides a supplement for regulatory network investigation.

Using the prediction data, a massive and complex network was built for CRC, which could be subdivided into 3 exclusive subnetworks, namely composite-SNW, miRNA-SNW, TF-SNW. We found that some components participated in three types of subnetworks simultaneously, including 15 genes (*FZD3*, *KCNA4*, *RAD21*, *KIAA1109*, *LYST*, *SCN11A*, *AKAP6*, *PCDHA13*, *ADAMTSL3*, *PCDH11X*, *MAP2K4*, *COL11A1*, *FBN1*, *NAV3* and *FN1*), 7 miRNAs (hsa-miR-25, hsa-miR-29a, hsa-miR-34a, hsa-let-7c, hsa-let-7e, hsa-miR-27b, hsa-miR-27a) and 8 TFs (FOXG1, TCF12, FOXJ2, MYCN, TFEB, CREB1, RUNX1, CBFB). In this study, we aimed to find out some significant components (miRNA, gene, or TF), which could serve as biomarker for the diagnosis, treatment, and prognosis of CRC. Although there were some interesting findings in the predictive network, it was difficult and unconvincing to determine significant components for the two reasons. First, the networks involved a great many components, especially TF-SNW, the regulations were massive and complex. Second, since the regulations involved in current networks were on the basis of multiple data sources, not all of which was validated by experiments, there might be some false positives. To improve our network, we integrated the expression data from TCGA into our analysis and used the co-expression to wash the unreliable regulations in the network. We then applied the hub identification to the concise network to determine significant components, whereby two miRNAs (hsa-miR-25 and hsa-miR-31), two genes (*ADAMTSL3* and *AXIN1*) and one TF (BRCA1) were identified significantly. Some of those genes, miRNAs and TFs have been reinforced by previous studies. To investigate values of these components on prognosis, we further analyzed association between their expression levels and survivals. We found that all of five components showed a promising predictive ability for CRC patients’ survival. For instance, low expression of hsa-miR-25 was observed with the increasing all-caused death risk for CRC patients. This is consistent with previous reports. In Li’s study, miR-25 was found to be down-regulated in human colon cancer tissues when compared to those in matched non-neoplastic mucosa tissues [56]. Functional studies revealed that restoration of miR-25 expression inhibited cell proliferation and migration. In contrast, miR-25 inhibition could promote the proliferation and migratory ability of cells. Stable over-expression of miR-25 also suppressed the growth of colon cancer-cell xenografts in vivo [57]. In Koo BH’s study, identification of frequent *ADAMTSL3* mutations in colorectal cancer suggested it might have a regulatory role in cellular homeostasis in colorectal epithelium or in pathways to colorectal malignancy [58]. In current study, the expression level ADAMTSL3 was found correlated with all-caused survival. Approximately half of the genes, miRNAs, TFs we predicted to be key roles had been studied and found to be associated with the regulation mechanism in CRC. These results indicated that the comprehensive CRC-specific regulatory network could provide valuable clues for researchers to identify critical CRC-related components. Furthermore, as hsa-miR-25 and *ADAMTSL3* had been proved playing important roles in CRC, but their exact interaction mechanism have not been clarified yet. Other significant components identified in our analysis also remain unclarified and need to explore by further researches.

A recent study by Fu et al. used a combinatorial strategy to identify CRC-related miRNA-mRNA pairs [59]. This study applied microarray expression data to identify dysregulated miRNAs and mRNAs, followed by anti-correlation computation and target relationship prediction based on TargetScan and miRanda. 72 miRNA-mRNA pairs were captured by including 22 miRNAs and 58 mRNAs. But these results were only limited in the binary regulation model between miRNAs and mRNAs, and the sample size of study was small (8 pairs). Although several studies aiming to uncover the regulation system of TFs and miRNAs have been reported [59–61], none have considered the integration of predictive data and experimental data in the application of an FFL model in CRC, improving the stability and reliability of the regulatory network. The process in current study could be a useful method and complement for revealing the complex regulation in other disease.

There also exist several limitations to our analysis. First, the number of relationship and its collective bias in the databases might make a potential effect on the final network construction and following significant components identification. In our analysis process, data selection were performed by multisource to reduce such impact. Second, as opposed to gene and miRNA, TF was not pre-selected to be CRC-related, which might influence the topology observation. In addition to the criteria used in regulation prediction in current study, more effective selection need to apply to CRC-related TF identification.

## Conclusions

Recently, network analyses have been applied to many diseases to reveal the complicated mechanisms and try to find out new makers or targets for the diagnoses and treatments. However, network analysis have not been systematically applied in colorectal cancer (CRC). In our paper, we build a systematic, comprehensive and complicated network for CRC, and finally through topologic analysis, we find some key miRNAs and feed forward loops that possibly play important roles in the regulation of CRC for further experiment design.

Furthermore, current FFL studies mostly rely on the predicted regulation information, which may lead to false positive outcomes. So some strategies are urgently needed to reduce the false positive rate. In this field, we integrated the predictive information and experimental co-expression data of TCGA project. We finally extracted significant components for CRC from a comprehensive and complex network using this strategy, which was confirmed in the subsequent prognosis analysis. This innovative strategy can be an inspiration for further researches in this field.

## Additional files

Additional file 1: Figure S1. Shows the degree distributions of nodes in the significant FFLs. (ZIP 289 kb)

Additional file 2: Tables S1 through S9. **Table S1.** Shows the CRC-related genes compiling from four sources. **Table S2.** shows the CRC-related miRNAs compiling from three sources. **Table S3.** shows merged 3-node FFLs including TF-FFLs, miRNA-FFLs and composite-FFLs. **Table S4.** shows the regulation information of the CRC-specific miRNA-TF mediated regulatory network. **Table S5.** shows the degree distribution of all nodes in the miRNA-SNW, TF-SNW and composite-SNW. **Table S6.** shows the regulation information of the CRC-specific significant FFLs. **Table S7.** shows the degree distribution of all nodes in the CRC-specific significant FFLs. **Table S8.** shows the miRNA targets predicted by using TargetScan. **Table S9.** shows the miRNA targets predicted by using miRanda. (ZIP 62739 kb)

Additional file 3: Texts S1 though S2. **TextS1.** Compiles CRC-related genes from multiple datasets. **Text S2.** compiles CRC-related miRNAs from multiple datasets. (ZIP 9 kb)

## Abbreviations

CRC: Colorectal cancer
FBL: feedback loop
FFLs: feed-forward loops
miRNA: microRNA
SNPs: single nucleotide polymorphisms
SNW: subnetwork
TCGA: The Cancer Genome Atlas
TFs: transcription factors
