# Supplementary figures and images for: Investigating MicroRNA and transcription factor co-regulatory networks in colorectal cancer
Source: BMC Bioinformatics. 2017 Sep 2;18:388. doi: 10.1186/s12859-017-1796-4 (PMC5581471; doi:10.1186/s12859-017-1796-4)

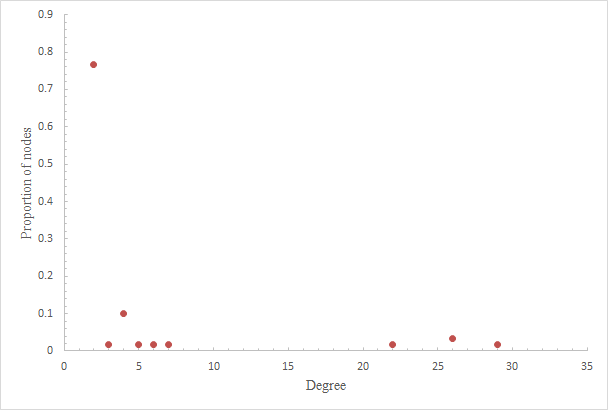

Supplement: Supplementary file 1 — Shows the degree distributions of nodes in the significant FFLs. (ZIP 289 kb) [file 12859_2017_1796_MOESM1_ESM.zip › Additional file 1 - Figures S1/S1_Fig.tif]
