# Supplementary material for: Investigating MicroRNA and transcription factor co-regulatory networks in colorectal cancer
Source: BMC Bioinformatics. 2017 Sep 2;18:388. doi: 10.1186/s12859-017-1796-4 (PMC5581471; doi:10.1186/s12859-017-1796-4)
Supplement: Supplementary file 3 — TextS1. Compiles CRC-related genes from multiple datasets. Text S2. compiles CRC-related miRNAs from multiple datasets. (ZIP 9 kb) [file 12859_2017_1796_MOESM3_ESM.zip › Additional file 3 – Texts S1 though S2/S1_Text.docx]

# Text S1. Compiling CRC-related genes from multiple datasets

**Genes from the Online Mendelian Inheritance in Man (OMIM)**

We searched the database OMIM [1] using the key word “colorectal cancer” and downloaded the results on November 11^th^ 2014. Records matching the condition of “phenotype mapping key ≥ 3” were extracted from the results downloaded before. Finally 25 relative genes were obtained.

**Genes from Cancer Gene Census (CGC)**

We downloaded the gene Excel file from the Cancer Gene Census (CGC), the Catalogue of Somatic Mutations in Cancer (COSMIC) [2] on November 11^th^ 2014. 22 CRC-related genes were extracted from CGC using the keywords “colorectal cancer or colon cancer”.

**Genes from a research by** [**Cancer Genome Atlas Network**](http://www.ncbi.nlm.nih.gov/pubmed/?term=Cancer%20Genome%20Atlas%20Network%5BCorporate%20Author%5D)**named *Comprehensive molecular characterization of human colon and rectal cancer*** [3]

Previously, [Cancer Genome Atlas Network](http://www.ncbi.nlm.nih.gov/pubmed/?term=Cancer%20Genome%20Atlas%20Network%5BCorporate%20Author%5D)’s research identified 32 somatic mutated genes (defined by MutSig11 and manual curation) in the hypermutated and non-hypermutated colon and rectal cancers. There were 15 and 17, respectively, in the hypermutated and non-hypermutated cancers. Two of them were both in hypermutated and non-hypermutated cancers, so 30 unique genes were obtained finally.

**Genes from** **a research by Kandoth C named *Mutational landscape and significance across 12 major cancer types*** [4]

This research studied 12 major cancer types and one of them was CRC. Through their analysis, 127 genes were involved in a wide range of cellular processes, and tended to display higher mutation frequencies above background. 23 in those 127 genes correlated with CRC were obtained.

**Summary of the genes from the four mentioned sources**

Summing up the genes mentioned before, 70 unique genes were obtained (Fig 1 in S1 Text and S10 Table).

**Genes from TCGA mutation data**

We calculated the gene mutation frequencies, which compared the colorectal cancer tissues with the normal ones based on TCGA mutation data [3] on November 11^th^ 2014. The top 1000 genes ranked by the mutation frequency were selected to overlap with the 70 genes mentioned before to find out the plateau of the increasing intersection. According to the variation of the number of overlap genes (Fig 2 in the Text S1), we selected the point (x: 380, y: 25) as the best balance point where the increasing rate of the overlap genes number increased no more and the top 380 genes from TCGA overlapped with the 70 genes, 25 of them were intersected. Considering that the
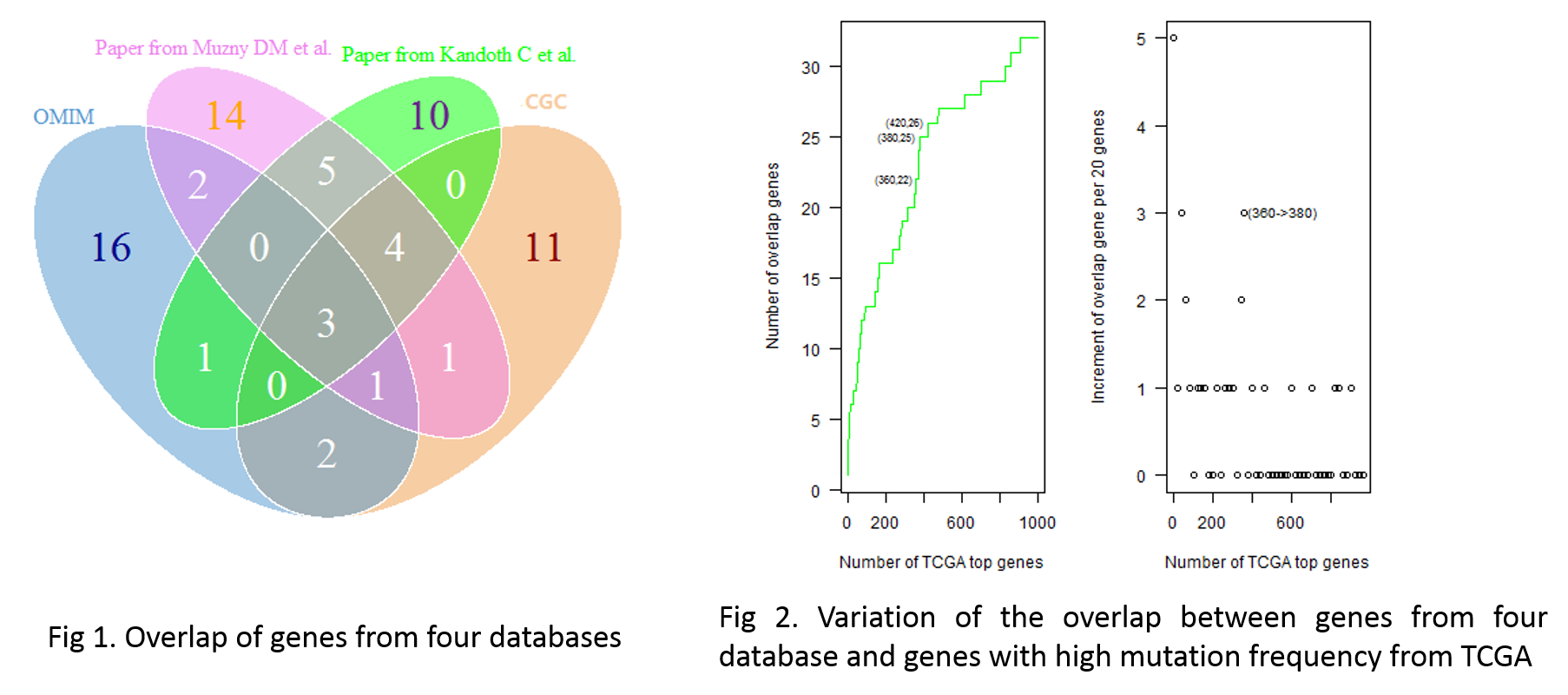
genes from top 380 to top 419 have the same mutation frequencies, which indicated that they might show small differences in the significance involved in the CRC development, 419 relative genes from TCGA were obtained for further analysis.

**Union of genes from TCGA and the 70 genes**

Summing up the 419 genes from TCGA mutation data and the mentioned 70 genes, 464 unique genes were obtained, which were used for further analysis.

References

1. Amberger J, Bocchini CA, Scott AF, Hamosh A (2009) McKusick's Online Mendelian Inheritance in Man (OMIM). Nucleic Acids Res 37: D793-796.

2. Forbes SA, Bindal N, Bamford S, Cole C, Kok CY, et al. (2011) COSMIC: mining complete cancer genomes in the Catalogue of Somatic Mutations in Cancer. Nucleic Acids Res 39: D945-950.

3. [Cancer Genome Atlas Network](http://www.ncbi.nlm.nih.gov/pubmed/?term=Cancer%20Genome%20Atlas%20Network%5BCorporate%20Author%5D) (2012) Comprehensive molecular characterization of human colon and rectal cancer. Nature 487: 330-337.

4. Kandoth C, McLellan MD, Vandin F, Ye K, Niu B, et al. (2013) Mutational landscape and significance across 12 major cancer types. Nature 502: 333-339.
