# Supplementary material for: Investigating MicroRNA and transcription factor co-regulatory networks in colorectal cancer
Source: BMC Bioinformatics. 2017 Sep 2;18:388. doi: 10.1186/s12859-017-1796-4 (PMC5581471; doi:10.1186/s12859-017-1796-4)
Supplement: Supplementary file 3 — TextS1. Compiles CRC-related genes from multiple datasets. Text S2. compiles CRC-related miRNAs from multiple datasets. (ZIP 9 kb) [file 12859_2017_1796_MOESM3_ESM.zip › Additional file 3 – Texts S1 though S2/S2_Text.docx]

# Text S2. Compiling CRC-related miRNAs from multiple datasets

**MiRNAs from MiR2Disease**

We downloaded the file named All Entries (txt file) from miR2Disease [1] on Sep 25^th^ 2014. 155 records were extracted from this file using the keyword “colorectal cancer” to search in the “Disease” column. Finally, 89 unique miRNAs were obtained from the 155 records (Table S11).

**MiRNAs from PhenomiR2.0**

We downloaded the database PhenomiR2.0 [2] on Sep 25^th^ 2014. 259 records were extracted from this database using the keyword “colorectal cancer” to search in the “Disease” column. Finally, 150 unique miRNAs were obtained from the 259 records (Table S11).

**MiRNAs from HMDD2.0**

We downloaded the database HMDD2.0 [3] on Sep 25^th^ 2014. 513 records were extracted from this database using the combination “Colorectal Neoplasms or Colonic Neoplasms” to search in the “disease” column. Finally, 211 unique miRNAs were obtained from the 513 records (Table S11).

**Identification of differentially expressed miRNA from miR2Disease, PhenomiR2.0, HMDD2.0**

The expressions of miRNAs from miR2Disease and PhenomiR2.0 have already been marked but HMDD2.0 not. For HMDD2.0, we downloaded the full papers through the related PubMed ID and read those texts to identify the reports including the expression comparison between CRC and normal controls, and we got 432 differentially expressed miRNAs. From the 432 miRNAs, 192 unique miRNAs were obtained.

**CRC-related miRNAs without consideration of regulation**

257 unique miRNAs were obtained after summing up all the results from miR2Disease, PhenomiR2.0 and HMDD2.0.

**References**

1. Jiang Q, Wang Y, Hao Y, Juan L, Teng M, et al. (2009) miR2Disease: a manually curated database for microRNA deregulation in human disease. Nucleic Acids Res 37: D98-104.

2. Ruepp A, Kowarsch A, Schmidl D, Buggenthin F, Brauner B, et al. (2010) PhenomiR: a knowledgebase for microRNA expression in diseases and biological processes. Genome Biol 11: R6.

3. Lu M, Zhang Q, Deng M, Miao J, Guo Y, et al. (2008) An analysis of human microRNA and disease associations. PLoS One 3: e3420.
